# Good practices in perinatal care and breastfeeding protection during the first wave of the COVID-19 pandemic: a national situation analysis among BFHI maternity hospitals in Spain

**DOI:** 10.1186/s13006-021-00407-y

**Published:** 2021-08-28

**Authors:** Barbara Muñoz-Amat, Carmen Rosa Pallás-Alonso, María-Teresa Hernández-Aguilar

**Affiliations:** 1 Neonatal Intensive Care Department, 12 de Octubre University Hospital, Madrid, Spain; 2 Breastfeeding Clinical Unit Dr Peset University Hospital, Valencia, Spain

**Keywords:** Baby-friendly Hospital Initiative, COVID-19 pandemic, IHAN-Initiative for Humanizing Birth and Breastfeeding Care, Maternity care practices, Lactation support, SARS-CoV-2

## Abstract

**Background:**

Although the positive effects of good clinical quality standards in perinatal care and breastfeeding support for women, newborns and families have been already demonstrated, many of these practices were disrupted during the COVID-19 pandemic. The objective of this study was to analyse the impact of the COVID-19 pandemic on perinatal care and breastfeeding support practices offered by the Spanish maternity hospitals committed to the UNICEF Baby-friendly Hospital Initiative (BFHI), to women with and without COVID-19.

**Methods:**

Implementation of perinatal practices was assessed by a cross-sectional survey conducted in May 2020 using an online questionnaire. Comparison with pre-pandemic situation and level of commitment to BFHI practices was performed.

**Results:**

Response rate was 50% (58/116). Mothers with COVID-19 suffered greater restrictions in the practices compared to women without COVID-19, with lower rates of companion of choice during labour (84% vs 100%; *p* = 0.003), skin-to-skin contact (32% vs 52%; *p* = 0.04), rooming-in (74% vs 98%; *p* <  0.001), companion of choice during hospital stay (68% vs 90%; *p* = 0.006), and breastfeeding support (78% vs 94%; *p* = 0.02). Practices were significantly less prevalent in COVID-19 mothers compared to pre-pandemic situation. A lower accompaniment rate was observed in non-COVID-19 group during delivery (24% vs 47.9%; *p* <  0.01). Hospitals with higher commitment to BFHI practices reported higher rates of skin-to-skin contact (45.2% vs 10.5%; *p* = 0.01) and rooming-in (83.9% vs 57.9%; *p* <  0.05) in COVID mothers. Fewer restrictions were observed in hospitals located in the regions where the pandemic hit harder. In these regions there was a significantly higher level of BFHI commitment of the hospitals, but no significant differences were observed in the average size of the hospital. All the practices suffered even more restrictions during the first weeks of the pandemic.

**Conclusion:**

All mothers suffered restrictions in perinatal care during the COVID-19 pandemic. Women with COVID-19 infection suffered more restrictions in perinatal practices than women without infection. The degree of commitment to WHO-UNICEF perinatal quality standards, integrated into the BFHI, was associated with maintenance of good clinical practices.

## Background

Emotional support from a companion of choice during labour and the delivery process, immediate and uninterrupted skin-to-skin contact, breastfeeding initiation within one hour of birth, seamless mother-infant closeness, and breastfeeding support, are considered good clinical quality standards of care. Although the positive effects of these clinical practices for women, newborns and families have been already demonstrated [1], many of these practices were disrupted during the COVID-19 pandemic. The impact of the outbreak on global health, the scarcity of material and human resources and the attempt to prevent contagion led to strict patient isolation measures that were also applied in perinatal care [2]. In order to avoid the mother-infant separation regardless of the mother COVID-19 situation, the World Health Organization (WHO) recommended maintaining the good clinical quality standards in perinatal care from the beginning of the pandemic when adequate protective measures were implemented [3]. Although some associations worldwide followed the WHO recommendations [4–6], some others differed from them. They published alternative guidelines including mother-infant separation measures, avoidance of direct breastfeeding and normative caesarean as the elective way of deliver [7, 8].

Initially, Spanish guidelines supported by the Ministry of Health and other scientific societies, proposed mother-infant separation for women with COVID-19 [9]. Nevertheless, the Spanish non-for-profit association *IHAN-Initiative for Humanizing Birth and Breastfeeding Care* (IHAN)*,* and other organisations advocated for maintaining the WHO quality standards for perinatal care [10, 11]. Thus, later, the Ministry and most of Spanish associations aligned their recommendations with those of the WHO [12]. Similar to other countries, perinatal care was also affected in Spain as a result of these discrepancies and inadequate standard care practices were implemented in some maternity hospitals.

IHAN is a Spanish non-for-profit organisation that promotes the implementation of best quality standards of care to perinatal care practices, including the protection and support of breastfeeding, in maternity wards and primary healthcare centers, in Spain. IHAN is also responsible for the implementation of the BFHI. IHAN’s strategy includes counselling and support to healthcare centers, and BFHI accreditation in 4 phases (“*A 4D Path*”). Along this *Path*, maternity hospitals commit to changing practices and form a breastfeeding committee in *Phase 1D;* draw up their action plan and regulations in *Phase 2D*; in phase 3D they ensure the competencies of their professionals, agree on protocols, and establish monitoring systems; and finally, they demonstrate compliance with the quality requirements in an external evaluation in *Phase 4D*, after which they receive the BFHI award. Accreditation is renewed every 5 years [13]. Following the declaration of commitment in *Phase 1D,* the hospital’s BFHI leader is invited to participate in a virtual support network coordinated by *IHAN*. It was in this network that IHAN received the first alerts about changes, in protocols and guidelines, at odds with BFHI practices. It was the fact of finding out what was happening during the whole process that motivated this study.

The objective of this study was to analyse the impact of the COVID-19 pandemic, on the perinatal care and breastfeeding support practices offered to women with and without COVID-19 infection by the Spanish maternity hospitals committed to BFHI.

## Methods

### Design

Cross-sectional survey. In May 2020, an online questionnaire designed by the authors for the purpose of the study, was sent to the IHAN co-ordinator of each of the 116 Spanish BFHI maternity hospitals or those in the process of achieving the BFHI award (1D to 4D phases). The questionnaire was available for three weeks (10 to 31 May 2020) and four reminders were sent to the co-ordinators. Information was requested regarding current practices and restrictions in previous pandemic weeks. Exclusion criteria: Those centers that had referred women with COVID-19 or had no cases were excluded. *Dependent variables:* companion of choice during labour, companion of choice during delivery, immediate skin-to-skin contact after birth, breastfeeding in the first hour of life, rooming-in, companion of choice during hospital stay, breastfeeding support, and extra measures of breastfeeding support after discharge if discharged early. Information on these practices was requested in relation to three situations: mother with negative PCR for SARS-CoV-2 or no clinical suspicion of COVID-19 (henceforth *without COVID-19*), women with positive PCR for SARS-CoV-2 or clinical suspicion of COVID-19 (henceforth *with COVID-19*) who were asymptomatic or oligo-symptomatic (henceforth *mild COVID-19*) and women with moderate or severe COVID-19 (henceforth *severe COVID-19*). Information on practices that were implemented pre-pandemic, was requested in a subsequent addendum, in January 2020. Hospitals were also asked to indicate the “BFHI Steps” that were most at risk because of the pandemic.

#### Independent variables

*Hospital size,* hospitals were stratified in two groups according to their annual number of deliveries (cut-off point was set at 1500 deliveries per year), *level of commitment to BFHI practices* (two groups were established with cut-off point set at *Phase 2D* or more), and *the degree of impact of the pandemic* in the Autonomous Region (AR) where hospitals belonged, measured by the adjusted cumulative incidence published by the Spanish National Epidemiological Surveillance Network [14]. For the latter, the cut-off level was set at 190 cases/100,000 inhabitants.

### Analysis

For the purposes of the analysis, current practices were considered if the answer was “always” or “unless contraindicated for clinical reasons”. The variation of practices attributable to the pandemic was analysed comparing the practices in place in January 2020 with the practices in the weeks of the May 2020 survey. The variations in implementation in relation to the other variables described were also analysed.

A descriptive analysis of the categorical variables was performed using absolute and relative frequencies and comparisons were made using a two-tailed chi-square test, α set at 0.05. StatGraphics Centurion XVII version 17.0.16 was used for the analysis.

### Ethical considerations

The study was evaluated by a Clinical Research Ethics Committee. It verified that the assessment and the issuing of an opinion was not required.

## Results

The response rate was 50% (58/116). Eight centers that had referred women with COVID-19 (6/116) or had no cases (2/116) were excluded. Of the remaining 50 hospitals, 90% (45/50) were public, 50% (25/50) attended more than 1500 deliveries per year, and 62% (31/50) were Baby-Friendly or at Phase 2D or more. Sixty-four per cent (32/50) performed PCR tests for SARS-CoV-2 virus on all mothers at admission, and 100% (50/50) had perinatal care protocols in place during COVID-19 pandemic.

Table 1 compares the practices regarding maternal health status: women without COVID-19 vs. women with COVID-19. It can be observed that women with COVID-19 suffered more restrictions than women without COVID-19. Good practices were less prevalent after the pandemic compared to the month before the pandemic started in our country (Table 2). Table 3 compares current practices, related to the number of births and the level of BFHI commitment, in both groups, women with COVID-19 and without COVID-19. Table 4 shows current practices in relation to the regional impact of COVID-19. Fewer restrictions were observed in hospitals located in the regions where the pandemic hit harder. In these regions there was a significantly higher level of BFHI commitment of the hospitals (80% were BFHI accredited or ≥ *Phase 2D* vs 35% in the regions where the pandemic had lower impact; *p* <  0,01), but no significant differences were observed in the average size of the hospital.

**Table 1:** Implementation of best quality standards of care in labour, delivery, birth, and breastfeeding support for women without COVID-19 vs with mild COVID-19. BFHI maternity hospitals in Spain, May 2020

	Without COVID-19	With mild COVID-19	
	*N* = 50 n (%)	N = 50 n (%)	p
Companion of choice during labour	50 (100)	42 (84)	0.003
Companion of choice during delivery	12 (24)	9 (18)	0.06
Immediate skin-to-skin after birth	26 (52)	16 (32)	0.04
Breastfeeding in the first hour of life	25 (50)	18 (36)	0.15
Rooming-in	49 (98)	37 (74)	< 0.001
Companion of choice during hospital stay	45 (90)	34 (68)	0.006
Breastfeeding support	47 (94)	39 (78)	0.02
Follow-up if discharged early	45 (90)	39 (78)	0.10
Special breastfeeding support measures	18 (36)	20 (40)	0.68

*N* total responses, *n* hospitals that implemented the practice for all deliveries, excluding emergency caesarean section

**Table 2:** Implementation of best standards of perinatal care in women without COVID-19 vs with mild COVID-19. Pre-pandemic vs the COVID-19 pandemic, May 2020

	Without COVID-19	Pre-pandemic		Mild COVID-19	Pre-pandemic	
	n/N (%)	n/N (%)	p	n/N (%)	n/N (%)	p
Companion of choice during labour	50/50 (100)	48/48 (100)	1	42/50 (84)	48/48 (100)	0.003
Companion of choice during delivery	12/50 (24)	23/48 (47.9)	0.01	9/50 (18)	23/48 (47.9)	0.001
Immediate skin-to-skin contact after birth	26/50 (52)	30/48 (62.5)	0.29	16/50 (32)	30/48 (62.5)	0.002
Breastfeeding in the first hour of life	25/50 (50)	33/48 (68.8)	0.59	18/50 (36)	33/48 (68.8)	< 0.001
Rooming-in	49/50 (98)	48/48 (100)	0.32	37/50 (74)	48/48 (100)	< 0.001
Companion of choice during hospital stay	45/50 (90)	47/48 (97.9)	0.10	34/50 (68)	47/48 (97.9)	< 0.001
Breastfeeding support	47/50 (94)	48/48 (100)	0.08	39/50 (78)	48/48 (100)	< 0.001

*N* Total responses, *n* hospitals that implemented the practice for all deliveries, excluding emergency caesarean sections

**Table 3:** Comparison of best perinatal care practices implemented in the first wave of the COVID-19 pandemic, by annual number of deliveries and level of hospital commitment to BFHI, for women without COVID-19, with mild COVID-19, and with severe COVID-19. BFHI maternity hospitals in Spain, May 2020

**Women without COVID-19**						
	**Annual births**			**Level of commitment to BFHI**		
	**< 1500**	≥ **1500**		**Phase < 2D**	**Phase** ≥ **2D**	
	*N* = 25 n (%)	N = 25 n (%)	*p*-value	*N* = 19 n (%)	*N* = 31 n (%)	*p*-value
Companion of choice during labour	25 (100)	25 (100)	1	19 (100)	31 (100)	1
Companion of choice during delivery	6 (24)	6 (24)	1	2 (10.5)	10 (32.3)	0.08
Immediate skin-to-skin contact after birth	16 (64)	10 (40)	0.004	7 (36.8)	19 (61.3)	0.09
Breastfeeding in the first hour of life	16 (64)	9 (36)	0.04	9 (47.4)	16 (51.6)	0.77
Rooming-in	25 (100)	24 (96)	0.31	19 (100)	30 (96.8)	0.43
Companion of choice during hospital stay	23 (92)	22 (88)	0.64	17 (89.5)	28 (90.3)	0.92
Breastfeeding support	22 (88)	25 (100)	0.07	16 (84.2)	31 (100)	0.02
Follow-up if discharged early	24 (96)	21 (84)	0.16	16 (84.2)	29 (93.5)	0.28
Special breastfeeding support measures	11 (44)	7 (28)	0.24	6 (31.6)	12 (38.7)	0.61
**Women with mild COVID-19**						
	**Annual births**			**Level of commitment to BFHI**		
	**< 1500**	≥ **1500**		**Phase < 2D**	**Phase** ≥ **2D**	
	N = 25 n (%)	N = 25 n (%)	p-value	N = 19 n (%)	N = 31 n (%)	p-value
Companion of choice during labour	21 (84)	21 (84)	1	15 (78.9)	27 (87.1)	0.44
Companion of choice during delivery	5 (20)	4 (16)	0.71	2 (10.5)	7 (22.6)	0.28
Immediate skin-to-skin contact after birth	11 (44)	5 (20)	0.07	2 (10.5)	14 (45.2)	0.01
Breastfeeding in the first hour of life	13 (52)	5 (20)	0.02	5 (26.3)	13 (41.9)	0.26
Rooming-in	18 (72)	19 (76)	0.75	11 (57.9)	26 (83.9)	0.04
Companion of choice during hospital stay	17 (68)	17 (68)	1	10 (52.6)	24 (77.4)	0.07
Breastfeeding support	20 (80)	19 (76)	0.73	12 (63.2)	27 (87.1)	0.047
Follow-up if discharged early	23 (92)	16 (64)	0.016	15 (78.9)	24 (77.4)	0.90
Special breastfeeding support measures	11 (44)	9 (36)	0.56	5 (26.3)	15 (48.4)	0.12
**Women with severe COVID-19**						
	**Annual births**			**Level of commitment to BFHI**		
	**< 1500**	≥ **1500**		**Phase < 2D**	**Phase** ≥ **2D**	
	*N* = 22 n (%)	N = 25 n (%)	p-value	*N* = 18 n (%)	*N* = 29 n (%)	p-value
Companion of choice during labour	15 (68.2)	17 (68)	0.99	11 (61.1)	21 (72.4)	0.42
Companion of choice during delivery	2 (9.1)	4 (16)	0.48	1 (5.5)	5 (17.2)	0.24
Milk expression encouraged and supported	17 (77.3)	20 (80)	0.82	11 (61.1)	26 (89.7)	0.02

N: Total responses, n: hospitals that implemented the practice for all deliveries, excluding emergency caesarean sections. Some questions were not responded by all hospitals

**Table 4:** Comparison of best perinatal care practices, being implemented in the first wave of the COVID-19 pandemic, by cumulative incidence of the disease in the Autonomous Region where hospitals were located, for women without COVID-19, with mild COVID-19, and with severe COVID-19. BFHI maternity hospitals in Spain, May 2020

**Women without COVID-19**			
	**CI > 190/100000**	**CI < 190/100000**	
	*N* = 30 n (%)	*N* = 20 n (%)	p-value
Companion of choice during labour	30 (100)	20 (100)	1
Companion of choice during delivery	9 (30)	3 (15)	0.22
Immediate skin-to-skin after birth	17 (56)	9 (45)	0.42
Breastfeeding in the first hour of life	14 (46.7)	11 (55)	0.56
Rooming in	29 (96.7)	20 (100)	0.41
Companion of choice during hospital stay	28 (93.3)	17 (85)	0.33
Breastfeeding support	29 (96.7)	18 (90)	0.33
Follow-up if discharged early	27 (90)	18 (90)	1
Special breastfeeding support measures	13 (43.3)	5 (25)	0.18
**Women with mild COVID-19**			
	**CI > 190/100000**	**CI < 190/100000**	
	N = 30 n (%)	N = 20 n (%)	p-value
Companion of choice during labour	28 (93.3)	14 (70)	0.03
Companion of choice during delivery	7 (23.3)	2 (10)	0.23
Immediate skin-to-skin after birth	14 (46.7)	2 (10)	0.006
Breastfeeding in the first hour of life	14 (46.7)	4 (20)	0.05
Rooming in	26 (86.7)	11 (55)	0.01
Companion of choice during hospital stay	25 (83.3)	9 (45)	0.004
Breastfeeding support	22 (73.3)	12 (60)	0.32
Follow-up if discharged early	23 (76.7)	16 (80)	0.78
Special breastfeeding support measures	16 (53.3)	4 (20)	0.02
**Women with severe COVID-19**			
	**CI > 190/100000**	**CI < 190/100000**	
	*N* = 27 n (%)	N = 20 n (%)	p-value
Companion of choice during labour	23 (85.2)	9 (45)	0.003
Companion of choice during delivery	5 (18.5)	1 (5)	0.17
Support for milk extraction	25 (92.6)	13 (65)	0.02

CI: Cumulative incidence as reported by National Epidemiologic Vigilance Network. n = hospitals implementing the practice for all deliveries, excluding emergency caesarean sections. Some questions were not responded by all hospitals

All practices suffered even more restrictions during the first weeks of the pandemic. More than one third of hospitals claimed to have further restricted the presence of a companion of choice during labour (34%; 17/50) or delivery (32%;16/50), immediate skin-to-skin contact after birth (36%; 18/50), breastfeeding in the first hour of life (38%;19/50), and breastfeeding support (46%, 23/50). These restrictions were more frequent in hospitals with > 1500 deliveries per year, with significant differences for immediate skin-to-skin contact after birth (56% vs. 16% *p* <  0.01), breastfeeding in the first hour of life (56% vs. 20%; p <  0.01), and breastfeeding support (60% vs. 32%; *p* <  0.05). A greater number of advancements towards BFHI accreditation or the higher degree of impact of the pandemic were not significantly associated with more restrictions in the first weeks, for any practice. Up to 80% of the participants considered that many restrictions could have been avoided, had more resources (more personnel, more and better personal protective equipment and more single-family rooms) been available.

Hospitals were asked to indicate the “Steps” that were most at risk because of the pandemic. “Step 2: Healthcare professionals must have the competencies to implement the practices” (37/50; 74%), followed by “Step 3: pregnant mothers must receive adequate information on the benefits and management of breastfeeding” (29/50; 58%), and “Step 10: breastfeeding support must be ensured after discharge” (26/50; 52%) were the most commonly reported.

## Discussion

This study analyses the impact of the COVID-19 pandemic on the implementation of quality standards for perinatal care including breastfeeding protection and support [1], in Spanish hospitals committed to BFHI. Our study shows that these practices were restricted during the first months of the pandemic in our country.

Confusion, overload and collapse of the entire hospital system characterised the first weeks of the pandemic [15]. Recommendations rapidly evolved alongside new knowledge and publications about SARS-CoV-2 virus, COVID-19 disease, and therapeutic tools to deal with it. A few weeks after the pandemic had started in China, data showed no evidence of vertical transmission, horizontal transmission risk seemed similar to that of general population, and no severe neonatal cases were reported [16, 17]. Despite this evidence, those for whom the fear of contagion outweighed the risk of loss of breastfeeding or mother-infant separation continued to recommend separation measures and discouraged direct breastfeeding [2, 9]. Meanwhile, based on the *primum-non-nocere* principle (first, do no harm), WHO and other scientific associations, recommended maintaining the standards of quality and humanisation for perinatal care and protecting breastfeeding considering the unlikely risks of vertical transmission or the neonatal infection through breast milk [3, 4]. In May, when this survey was completed, these recommendations had even greater backing due to the accumulated evidence in the previous months. The morbidity and mortality rates, in newborns and infants, were very low worldwide and no cases of vertical transmission, or via breast milk [17, 18] had been reported. Despite this knowledge, some social networks, press and a great number of scientific publications warned that the right to accompaniment during the labour and delivery process was being withdrawn in worldwide maternity hospitals, immediate skin-to-skin contact and early or direct breastfeeding were not allowed, and mothers and newborns were being separated in a critical period [2, 4].

The survey was launched when the situation in Spain had already substantially improved, hospitals were starting to improve their situation and care could begin to be planned and organised [15]. It was the right time to analyse actions and identify the areas that leave room for improvement. In their study, Perrine et al. involved 1344 of 2018 hospitals in a surveillance network and collected data on care in US maternity wards between July and August 2020 [19]. At that time, the epidemiological situation in the USA was similar to ours in April, but for the accumulated knowledge of the effects of the pandemic in Europe and Asia in the previous months. The proportion of maternity hospitals that stopped assisting women with COVID-19 was higher in Spain (10%) than in the USA (1.5%). Among those centers that maintained assistance, about 20% in both countries advised against mothers practicing immediate skin-to-skin contact. However, in Spain, 32% of the hospitals allowed immediate skin-to-skin without restrictions for women with mild COVID-19, compared to 13% in the USA. Rooming-in was more frequently allowed in Spanish maternity hospitals, where 26% separated mothers with mild COVID-19 from their babies, compared to 43.1% in American hospitals. Rates of early discharge from the maternity ward (before 48 h) were similar, around 75% in both studies. Although, in both countries, maternity hospitals acknowledged that they decreased their breastfeeding support, 78% of the Spanish facilities and 67% of the USA ones reported their support to breastfeeding. Breastfeeding in the first hour of life was allowed in only around one in three women both in Spain (36%) and the USA (33.3%) [19]. A total of 40% of the facilities in Spain, confirmed that they have implemented some strategies to mitigate breastfeeding rate decline. The differences described may be partially explained by the fact that the recommendations of our Ministry of Health and scientific societies were in line with those of the WHO, and that the maternity hospitals included in the Spanish study were also committed to the BFHI.

Our study is the first to analyse the impact of the COVID-19 pandemic on perinatal care in women without COVID-19. Our results show that, although these women, newborns and families, suffered fewer restrictions than those with COVID-19 infection, most of them were separated from their partners during the labour period and, in many cases at the time of delivery, as well.

The degree of commitment to WHO-UNICEF perinatal quality standards, integrated into the BFHI, was associated with increased maintenance of good practices, despite the pandemic. Moreover, we found that it was in the regions where the pandemic hit harder, where women suffered the least restrictions on their rights and where quality practices were most often maintained. Specifically, women in these regions were significantly more often able to have a companion during the labour, delivery and postpartum periods, to practice skin-to-skin contact and to room-in with their babies. The fact that there was a higher level of commitment among hospitals in these regions, reinforces the possibility that BFHI protected families from loss of quality perinatal care, even under very adverse conditions. On the other hand, our data, contrast with those of Parker in the USA, who did not find differences in care related to BFHI practices [20].

It is possible that skin-to-skin contact and breastfeeding in the first hour of life were more frequently restricted in hospitals with more than 1500 deliveries per year. It could be partially explained considering the higher clinical workload suffered by these hospitals. It is possible that smaller hospital suffered less clinical workload and, consequently, they could better reorganise their care strategy. A similar situation has been described in the USA, where restriction of skin-to-sin contact was more frequent in level 3–4 hospitals, although no differences were observed with the other practices [20].

Most of the hospitals in our study, considered that a better provision of resources could have avoided some restrictions. Similarly, professionals participating in the neonatal COVID-19 registry of the Spanish Neonatal Society (SENeo) reported that around 80% of the admissions of neonates born to mothers with COVID-19 infection were associated with more difficulties in the organisation of the hospital for mother-infant rooming-in [21]. This situation has also been described in the USA, where the shortage of protective equipment, material means and human resources significantly affected the quality of care for newborns and families [22].

Restrictions to labour and delivery accompaniment by a person of choice, prohibiting immediate skin-to-skin contact and early or direct breastfeeding, and postnatal separation of mother and infants, have caused to women, newborns and their families high level of distress and anxiety. Consequently, all these disruptions during the COVID-19 pandemic have caused a severe impact on the family and social relationships [23]. It is highly likely that this negative impact could cause serious short- and long-term consequences on women’s mental and physical health [23, 24] and on the development of a secure attachment and bonding [25]. In addition, a negative impact of these restrictions on breastfeeding has also been described, with lower breastfeeding rates observed at discharge, and months later, in mothers who were separated from their newborns [24]. It is important to remember that all these practices are considered quality standards in perinatal care because their protective impact have been measured and demonstrated on maternal and infant health [26]. The evidence accumulated in a pandemic year time shows that good perinatal care practices promoted by the BFHI, such as skin-to-skin contact, immediate breastfeeding and keeping mother and infants together, with adequate prevention measures, do not increase the risk of disease [27, 28]. On the contrary, separating mothers from their infants increase their risk of nosocomial infection [28], while IgA found in the milk of mothers with COVID-19 could provide extra protection for the neonate [29].

The main strength of this study is that it is the first nationwide analysis, in Europe, to measure the variation in maternity care practices, in the first wave of the COVID-19 pandemic. Furthermore, our study examined these variations of care both for women with and without COVID-19. Its main limitation lies in the fact that the survey was conducted among hospitals committed to BFHI and this fact may have led to an underestimation of the impact of the pandemic on some of the studied practices, since these maternity hospitals have implemented them better than the average. We were not able to report on the practices in the other 58 hospitals that did not respond to the survey.

## Conclusions

The COVID-19 pandemic undoubtedly had collateral effects, on the health of women and newborns, due to the restrictions on good practices in perinatal care. It seems that the commitment of professionals and institutions to BFHI has helped to protect families from the loss of quality in perinatal care, including protection of breastfeeding, while care was being reorganised to protect them from COVID-19 infection. These considerations may be relevant in dealing with unforeseen circumstances, in next waves of this pandemic or any other, that could affect birth care and breastfeeding support.

## Data Availability

The datasets used and/or analysed during the current study are available from the corresponding author on reasonable request.

## Abbreviations

AR: Autonomous Region
BFHI: Baby-friendly Hospital Initiative
CI: Cumulative incidence
COVID-19: Coronavirus disease 2019
IHAN: *Initiative for Humanizing Birth and Breastfeeding Care*
PCR: polymerase chain reaction
SARS-CoV-2: Severe acute respiratory syndrome coronavirus 2
SENeo: Spanish Neonatal Society
WHO: World Health Organization
